# DCGR: feature extractions from protein sequences based on CGR via remodeling multiple information

**DOI:** 10.1186/s12859-019-2943-x

**Published:** 2019-06-20

**Authors:** Zengchao Mu, Ting Yu, Enfeng Qi, Juntao Liu, Guojun Li

**Affiliations:** 1 School of Mathematics, Shandong University, Jinan, 250100 Shandong Province China; 2 College of Mathematics and Statistics, Guangxi Normal University, Guilin, 541001 China

**Keywords:** Protein feature extraction, CGR curve, Physicochemical property, Algorithm

## Abstract

**Background:**

Protein feature extraction plays an important role in the areas of similarity analysis of protein sequences and prediction of protein structures, functions and interactions. The feature extraction based on graphical representation is one of the most effective and efficient ways. However, most existing methods suffer limitations from their method design.

**Results:**

We introduce DCGR, a novel method for extracting features from protein sequences based on the chaos game representation, which is developed by constructing CGR curves of protein sequences according to physicochemical properties of amino acids, followed by converting the CGR curves into multi-dimensional feature vectors by using the distributions of points in CGR images. Tested on five data sets, DCGR was significantly superior to the state-of-the-art feature extraction methods.

**Conclusion:**

The DCGR is practically powerful for extracting effective features from protein sequences, and therefore important in similarity analysis of protein sequences, study of protein-protein interactions and prediction of protein functions. It is freely available at https://sourceforge.net/projects/transcriptomeassembly/files/Feature%20Extraction.

**Electronic supplementary material:**

The online version of this article (10.1186/s12859-019-2943-x) contains supplementary material, which is available to authorized users.

## Background

Similarity analysis of protein sequences plays an important role in protein sequence studies, e.g. the prediction or classification of protein structures and functions. In General, the biological function of a protein is determined by its three dimensional structure which is dependent on the linear sequence of amino acids. Rigden [1] presented that one of the fundamental principles of molecular biology is that proteins having similar sequences possess similar functions. Up to now, lots of methods have been proposed for the similarity analysis of protein sequences, among which the graphical representation of protein sequences is one of the most used and effective strategies [2–21].

The chaos game representation (CGR) based on an iterative function system was firstly proposed for the representation of DNA sequences by Jeffrey in 1990 [22]. The Jeffrey’s CGR is drawn within a quadrate with four vertices referring to nucleotides A, C, G and T. The first point is placed halfway between the center of the quadrate and the vertex corresponding to the first nucleotide of the sequence. The *i*-th (i > 1) point is placed halfway between the (*i*-1)-th point and the vertex corresponding to the *i*-th nucleotide. Being capable of discovering the inner pattern of gene sequences, CGR has been widely used in the investigation of DNA sequences [23–28]. Encouraged by the CGR of DNA sequences, the CGR of protein sequences has also been extensively studied by many researchers. Fisher et al. [29] first proposed an improved CGR of protein sequences, which was produced in a 20-side regular polygon with 20-vertices representing 20 kinds of amino acid. Randić et al. [30] constructed the CGR of protein sequences in the interior of a unit circle, on the circumference of which 20 amino acids are located uniformly according to the alphabet order of their three letter codes.

Amino acids themselves have physicochemical properties, which are important for protein structures, functions and protein-protein interactions and have strong effects on the pattern of protein evolution. Therefore, physicochemical properties of amino acids have been widely used in protein sequence studies, such as similarity analysis of protein sequences, prediction of protein subcellular localization and protein structural class prediction [2–15, 18–20, 31–38]. In [39], Randić mentioned that ordering amino acids based on their physicochemical properties may offer better insights in comparative studies of proteins than representations of proteins based on alphabetical ordering of amino acids, which is essentially equivalent to random ordering. Following Randić’s approach, He et al. [31, 40] proposed some different cyclic orders for the 20 amino acids to introduce the CGRs of protein sequences based on the physicochemical properties of amino acids. We denote the above CGRs by 20-CGR as 20 kinds of letters are used to represent protein sequences. Basu et al. [41] used a 12-sided regular polygon to generate the 12-CGR of protein sequences, each vertex of which represents a group of amino acids based on the conservative substitutions. Later Yu et al. [32] and Manikandakumar et al. [33] proposed 4-CGR, 5-CGR and 6-CGR for protein sequences, in which 4, 5 and 6 kinds of letters were used to represent protein sequences, respectively. In fact, using reduced amino acid alphabet to represent a protein sequence would easily result in loss of sequence information, since the amino acids belonging to the same group are considered identical.

So far, CGR method has achieved many applications in the studies of bioinformatics. The key issue in the application of CGR is to extract as many useful features as possible from CGR and several studies showed that those extracted features plays important roles in protein studies [25–28, 31, 34–38, 40–42]. One of the most frequently used feature extraction methods is the so-called FCGR, in which the CGR image is split into small grids and the frequencies of points falling into each grid are taken as the feature of the corresponding protein sequence. For example, in [34–38, 41], the CGR image of a protein sequence was split into 24 grids, and the frequencies of points falling into 24 grids are counted and taken as the numerical characteristics of the protein sequence. Under this procedure, a protein sequence can be converted into a 24-dimensional vector. Although FCGR method could effectively extract useful information from CGR, however, it loses the distribution information of the points in each grid, which is proved of great importance in this paper.

In this paper, we propose a novel feature extraction method of protein sequences based on the Randić’s 20-CGR, which effectively integrates the physicochemical properties of amino acids into the construction of CGR curves and makes full use of the distribution information of points for extracting numerical characteristics from CGR curves. When tested on five data sets, it performs much better than all the compared methods.

## Results

In this study, five most frequently used data sets were adopted to evaluate the performance of the new method DCGR in comparison with different feature extraction methods and also the sequence alignment method ClustalW.

### Similarity analysis of 9 ND5 protein sequences

We first apply DCGR to analyze the similarities of the ND5 protein sequences from 9 kinds of species (detailed in Additional file 1: Table S1), which have been widely used in different studies and considered as a standard to evaluate the model [2, 4–8, 10, 12–15, 19, 20, 31, 43].

Based on DCGR, we first obtained a 9 × 632 feature matrix for the 9 protein sequences. Then PCA was used to reduce the dimensionality of the feature vectors. Here, only the first 6 principal components were selected and therefore a 9 × 6 reduced feature matrix could be built. *Euclidean* distance was used to calculate the distance between each two protein sequences (see Additional file 1: Table S2 for the calculated distances between protein sequences). The smaller distance between two proteins, the closer relationship between the two species.

From Additional file 1: Table S2, it is clear that the distance between Fin whale and Blue whale is the smallest of all, demonstrating the closest phylogenetic relationship between them. The distances among Human, Pigmy chimpanzee, Common chimpanzee and Gorilla are also small, showing that they are also similar. In addition, we can also find that Rat and Mouse have a relatively close relationship. However, the distance between Opossum and any other 8 species was very large, demonstrating its far relationship with the others. All results are consistent with the known evolutionary relationship among the 9 species.

**Fig. 1:**
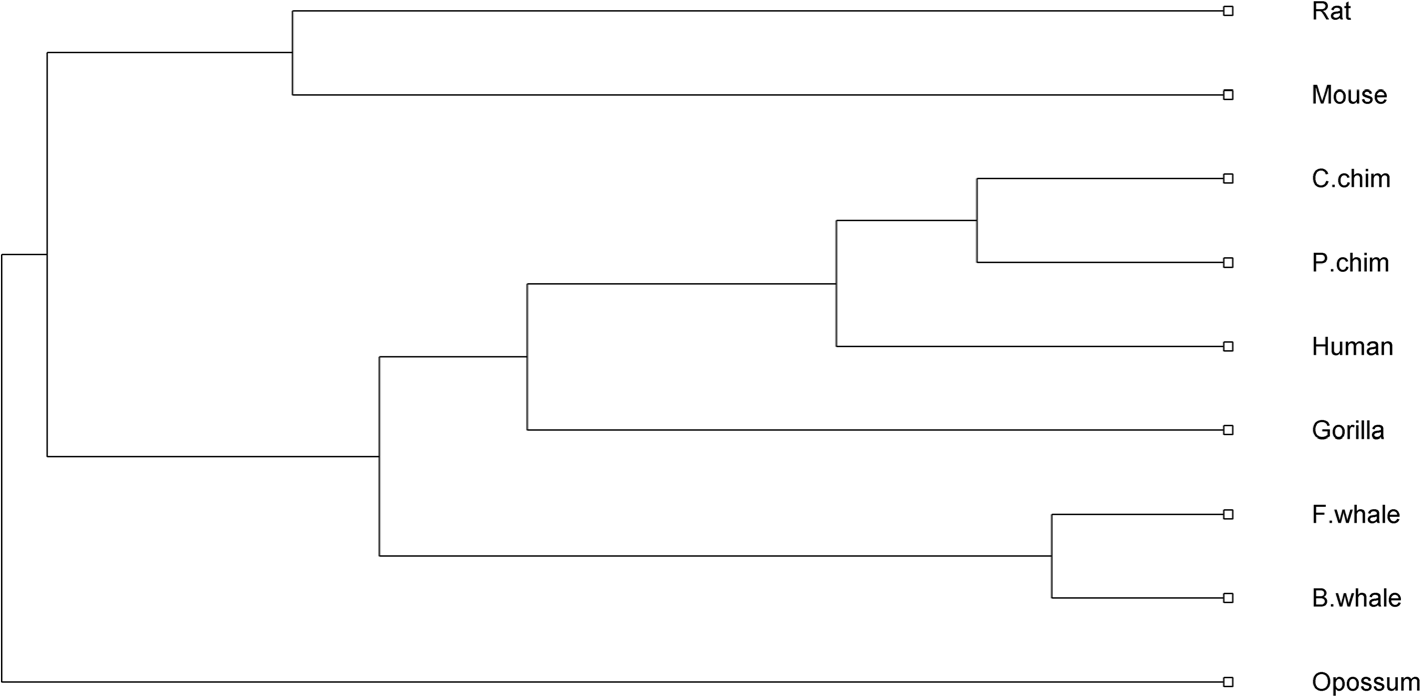
Phylogenetic tree of the nine ND5 proteins constructed by DCGR

For direct survey of evolutionary relationship among the 9 species, we construct the phylogenetic tree based on the distance matrix in Additional file 1: Table S2 shown in Fig. 1, which clearly illustrates four different branches clustered from the 9 species. The first branch consists of the Rodentia (Rat, Mouse), the second one the Primates (Pigmy chimpanzee, Common chimpanzee, Human, Gorilla), the third one the Cetacea (Fin whale and Blue whale) and the fourth one the Marsupialia (Opossum). ClustalW is one of the most popular multiple sequence alignment methods. Here, we also construct the phylogenetic tree by using ClustalW shown in Additional file 1: Figure S1, which shows very similar evolutionary relationships of the 9 species with our results.

### Similarity analysis of 36 protein sequences

**Fig. 2:**
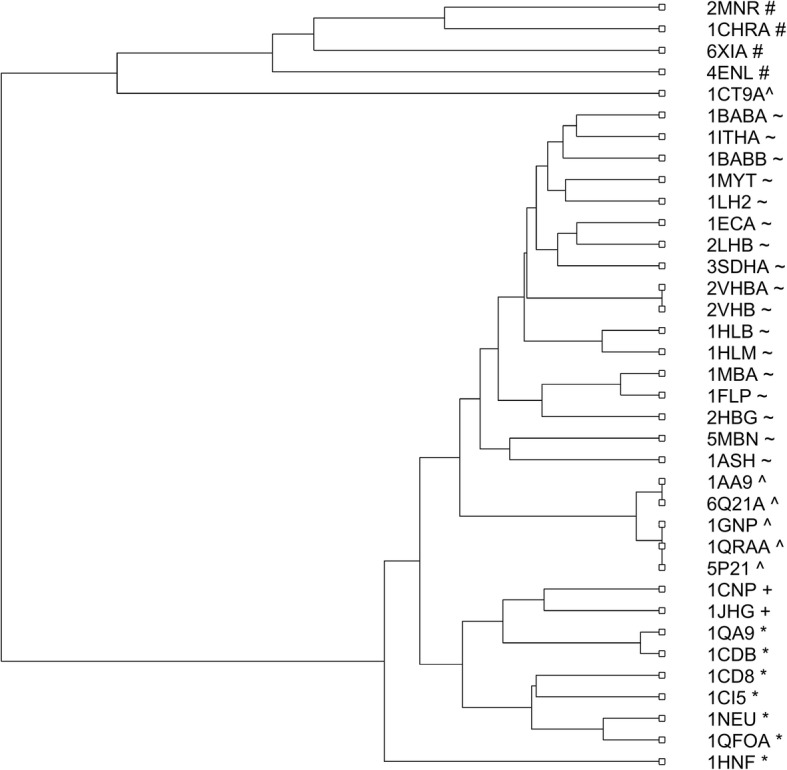
Phylogenetic tree of the 36 proteins constructed by DCGR

In the second example, we apply our method to analyze a data set consisting of 36 protein sequences of 5 different families: Globin (1eca, 5mbn, 1hlb, 1hlm, 1babA, 1babB, 1ithA, 1mba, 2hbg, 2lhb, 3sdhA, 1ash, 1flp, 1myt, 1lh2, 2vhbA, 2vhb), Alpha–Beta (1aa9, 1gnp, 6q21A, 1ct9A, 1qraA, 5p21), Tim-Barrel (6xia, 2mnr, 1chrA, 4enl), Beta (1 cd8, 1ci5, 1qa9, 1cdb, 1neu, 1qfoA, 1hnf), and Alpha (1cnp, 1jhg) [20, 43–48]. After extracting features by the method DCGR and reducing the dimensionality using PCA, the *Manhattan* distance was used to calculate the distance matrix of the 36 protein sequences. Similarly, we constructed the phylogenetic tree of the 36 protein sequences in Fig. 2, demonstrating that the 36 proteins have been accurately clustered into the 5 corresponding families, with only one erroneously clustered protein 1ct9.

In order to illustrate the superiority of DCGR, we compared its performance with six other methods including ClustalW in [20, 43–47], and the phylogenetic trees constructed by the six methods have been shown in Additional file 1: Figures S2-S8. After comparison, DCGR showed best performance since most of the six methods erroneously clustered at least three proteins, especially for ClustalW, which erroneously clustered 5 proteins as reported in [43].

### Similarity analysis of 50 beta-globin protein sequences

**Fig. 3:**
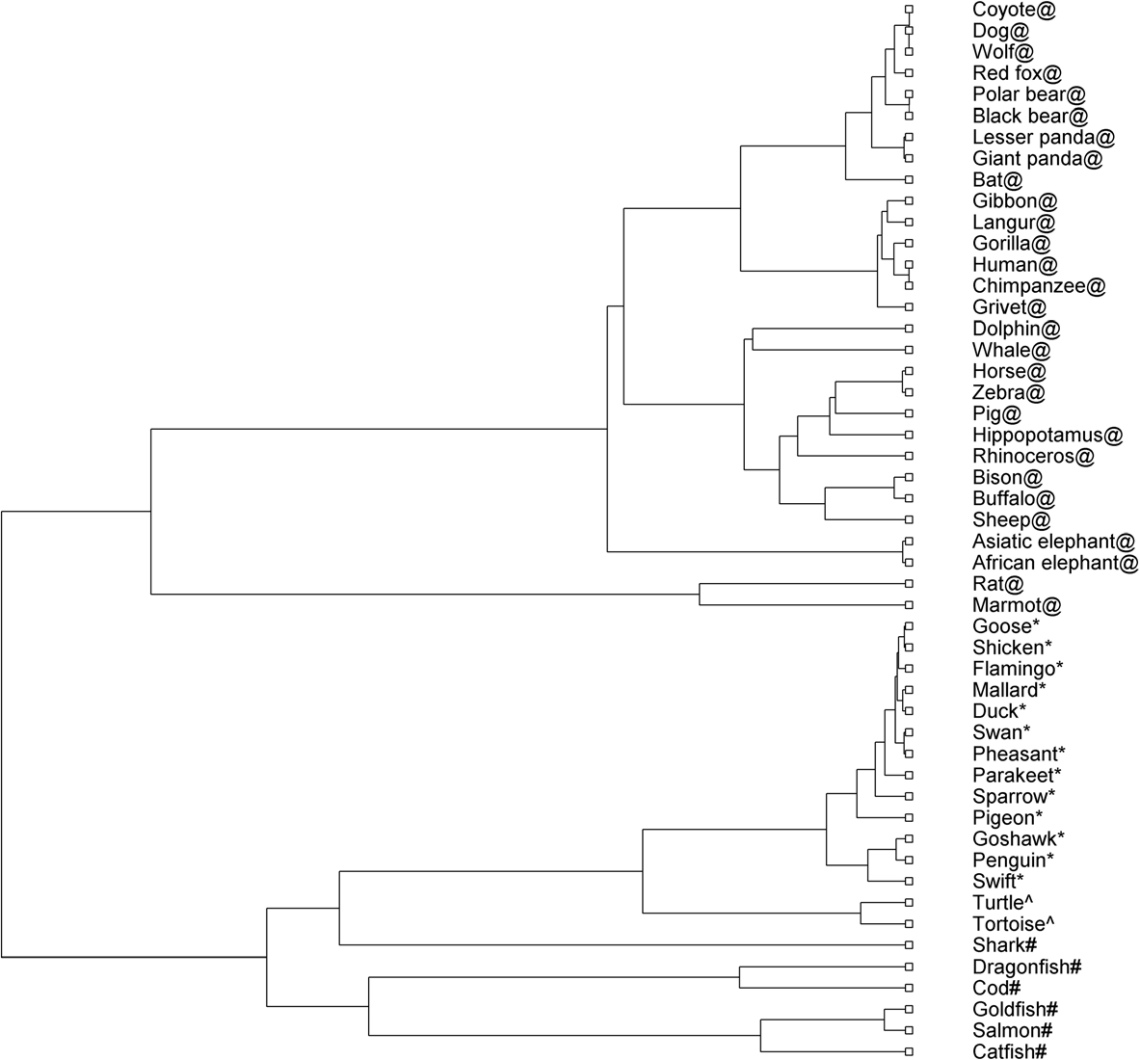
Phylogenetic tree of the 50 beta-globin protein sequences constructed by DCGR

This data set contains 50 beta-globin protein sequences from 50 species studied in [46, 49–53], and the accession numbers have been shown in Additional file 1: Notes 1.2. After extracting features by the method DCGR and reducing the dimensionality using PCA, the *Cosine* distance was used to calculate the distance matrix of 50 beta-globin protein sequences, and the phylogenetic tree was also constructed in Fig. 3.

As shown in Fig. 3, the 50 beta-globin protein sequences are correctly grouped into two clusters corresponding to mammals and non-mammals, respectively. For the mammal cluster, the beta-globin proteins belonging to Carnivora (Black bear, Lesser panda, Giant panda, Coyote, Wolf, Red fox, Dog, Polar bear), Primate (human, grivet, gorilla, langur, gibbon, and chimpanzee), Cetacea (Whale, Dolphin), Bovidae (Sheep, Bison, Buffalo), Proboscidea (Asiatic elephant, African elephant) and Rodentia (Rat, Marmot) are accurately separated and grouped into respective taxonomic classes. In addition, in the branch consisting of Artiodactyla and Perissodactyla, only the Rhinoceros is erroneously clustered. While for the non-mammal cluster, the beta-globin proteins belonging to aves, fish and reptile are also perfectly separated and grouped into respective taxonomic classes. In addition, for the proteins belonging to fishes, the chondrichthyes (Shark) is accurately separated from the actinopterygii (Dragonfish, Cod, Goldfish, Salmon and Catfish) as an independent branch, which is consistent with the known evolutionary relationships.

The phylogenetic trees of other methods [46, 49–53] including ClustalW have also been shown in Additional file 1: Figures S9-S15. After comparison, we found that ClustalW achieves very similar results with our method DCGR, while the other methods performs much worse since even the mammals and non-mammals cannot be correctly separated by the methods in [46, 49–53], and lots of proteins are erroneously clustered by the methods in [46, 51–53].

### Similarity analysis of 25 TFs

**Fig. 4:**
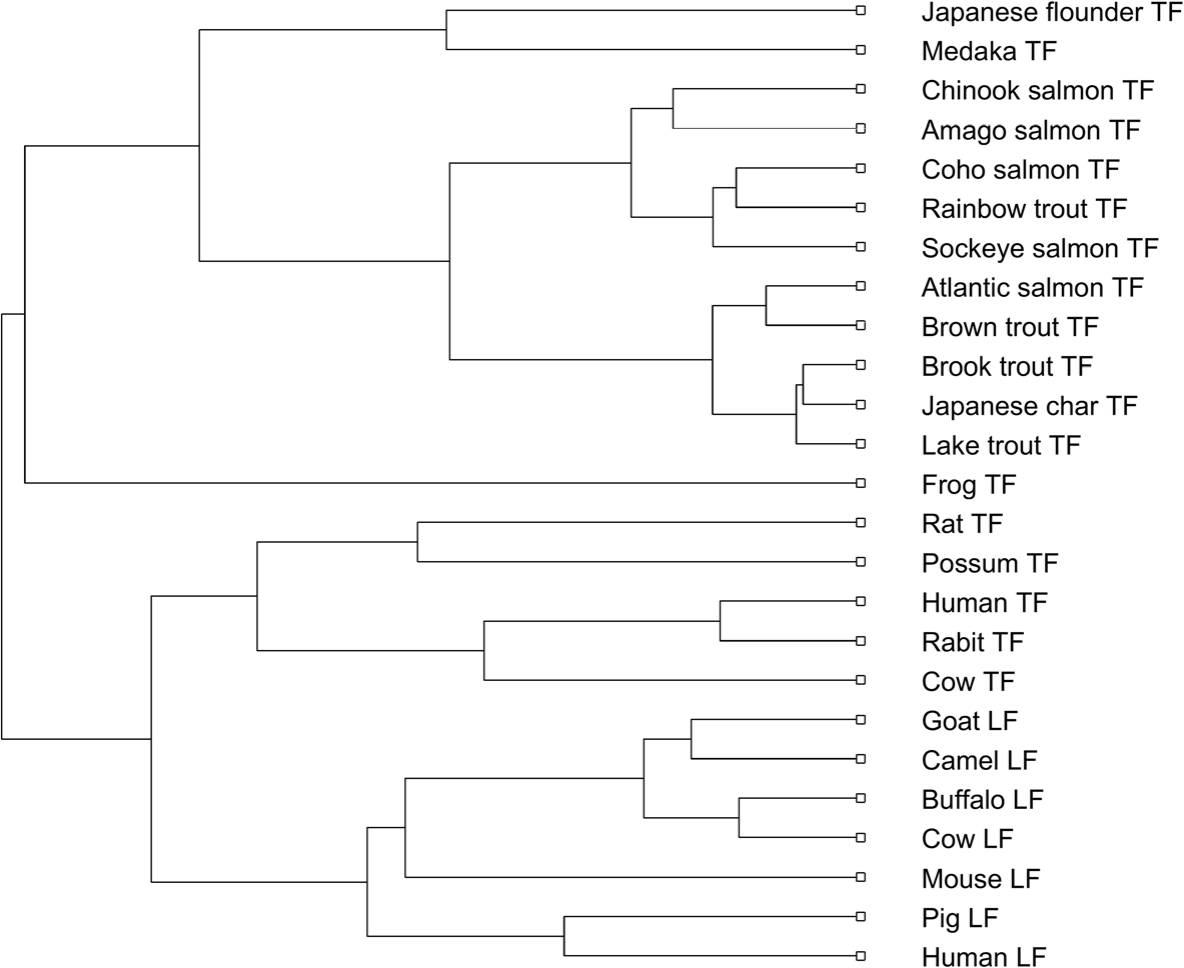
Phylogenetic tree of the 25 TFs constructed by DCGR

For this experiment, we select transferrin sequences from 25 vertebrates, which has been well studied by Ford [54]. Their taxonomic information and accession numbers are shown in Additional file 1: Table S3. Similarly processed by DCGR as before, the *Manhattan* distance was used to calculate the distance matrix of the 25 transferrin sequences, and the phylogenetic tree of the 25 TFs was also constructed in Fig. 4.

From Fig. 4, it is easy to find that all the sequences are accurately classified into the fish, amphibian and mammal groups. In the group of mammals, all the sequences belonging to transferrin (TF) proteins and lactoferrin (LF) proteins are also correctly separated and grouped into respective taxonomic classes. In the group of fishes, all the TFs from Salmonidae are clustered together and form a separate branch. In addition, the TFs belonging to Salmo (Atlantic salmon TF, Brown trout TF), Salvelinus (Lake trout TF, Brook trout TF, Japanese char TF) and Oncorhynchus (Chinook salmon TF, Coho salmon TF, Sockeye salmon TF, Rainbow trout TF, Amago salmon TF) are also correctly clustered and form separate branches, respectively. All these results are completely consistent with known evolutionary relationships. The phylogenetic tree constructed by DCGR is also great consistent with that obtained in [54] (see Additional file 1: Figure S16 for details), which is the most classical result among all the known. However, Possum TF is erroneously clustered in [54], which directly demonstrates that the DCGR is more reliable. For comparison, we also illustrated the phylogenetic tree constructed by ClustalW in Additional file 1: Figure S17, which shows similar results with our method DCGR.

### Similarity analysis of 27 AFPs

**Fig. 5:**
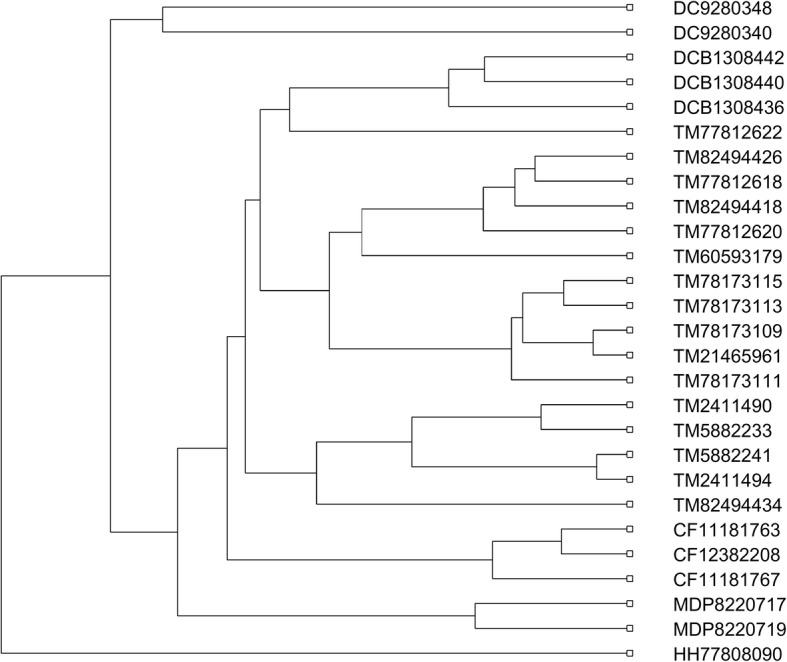
Phylogenetic tree of the 27 AFPs constructed by DCGR

For the last experiment, the 27 antifreeze protein sequences (AFPs) studied in [43, 52, 55] were used to evaluate the performance of our method. Antifreeze proteins are a class of proteins produced by certain vertebrates, plants, fungi and bacteria that permit their survival in subzero environments by binding to small ice crystals to inhibit growth and recrystallization of ice. The 27 AFPs were selected from Choristoneura fumiferana (CF), *Tenebrio molitor* (TM), *Hypogastrura harveyi* (HH), Dorcus curvidens binodulosus (DCB), Microdera dzhungarica punctipennis (MDP) and *Dendroides Canadensis* (DC), whose taxonomic information and accession numbers are provided in Additional file 1: Table S4. After feature extractions of the 27 AFPs by DCGR, the *standardized Euclidean* distance was used to calculate the distance matrix, and the phylogenetic tree of the 27 AFPs was constructed in Fig. 5. From Fig. 5, it clearly shows that the AFPs of the same species are accurately grouped together. In addition, the HH protein has a far relationship with each of the other 26 AFPs, which is consistent with the result in [56]. However, all the other compared methods [43, 52, 55] including ClustalW cannot accurately group all the proteins into respective taxonomic classes. The phylogenetic trees constructed by these methods have been shown in Additional file 1: Figures S18-S21. For example, ClustalW erroneously divided the TM proteins into two separate groups, while the methods in [43, 52, 55] failed separating the HH protein from the other ones.

We could therefore conclude from all these experiments that our method DCGR demonstrates significant superiority over all the state-of-the-art methods, and it even outperforms the method ClustalW, which is based on sequence alignment.

### Importance of the distribution information of points in the CGR image

Applying the distribution information of points in CGR image is a key step in the design of DCGR and makes an essential difference from the other FCGR methods. Traditional FCGR approaches first divide the CGR image into small grids and then take only the point frequency in each grid as numerical characteristics of the sequence without considering the distribution information of the points in each grid as in our method. In order to evaluate the importance of the distribution information of the points in the divided grids, we only took the point frequencies of the four segments as the numerical characteristics of the CGR curve and also used it to construct the phylogenetic trees of the above five data sets, respectively.

**Fig. 6:**
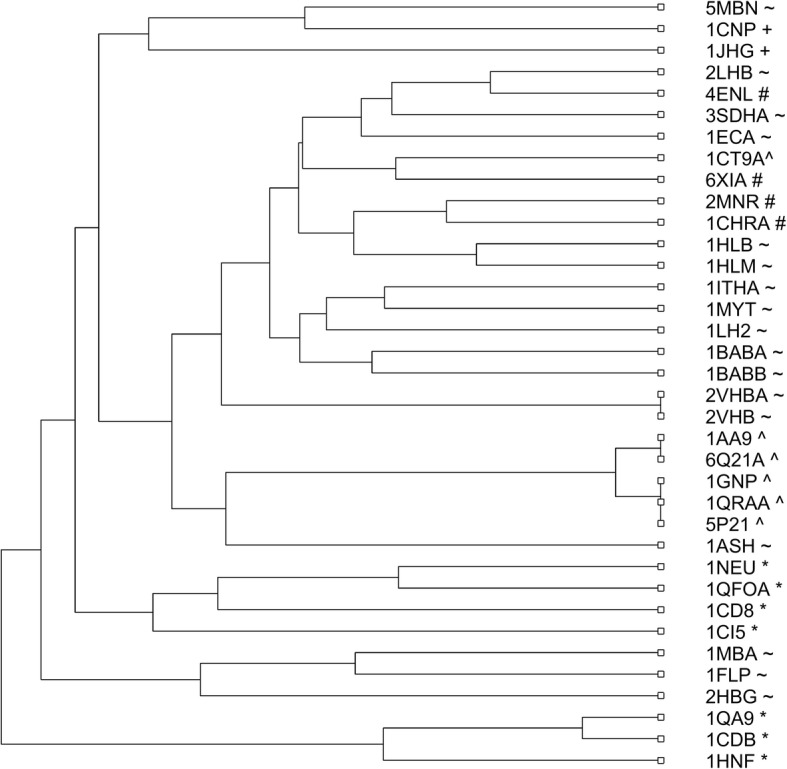
Phylogenetic tree of the 36 proteins constructed without considering the distribution information of points

**Fig. 7:**
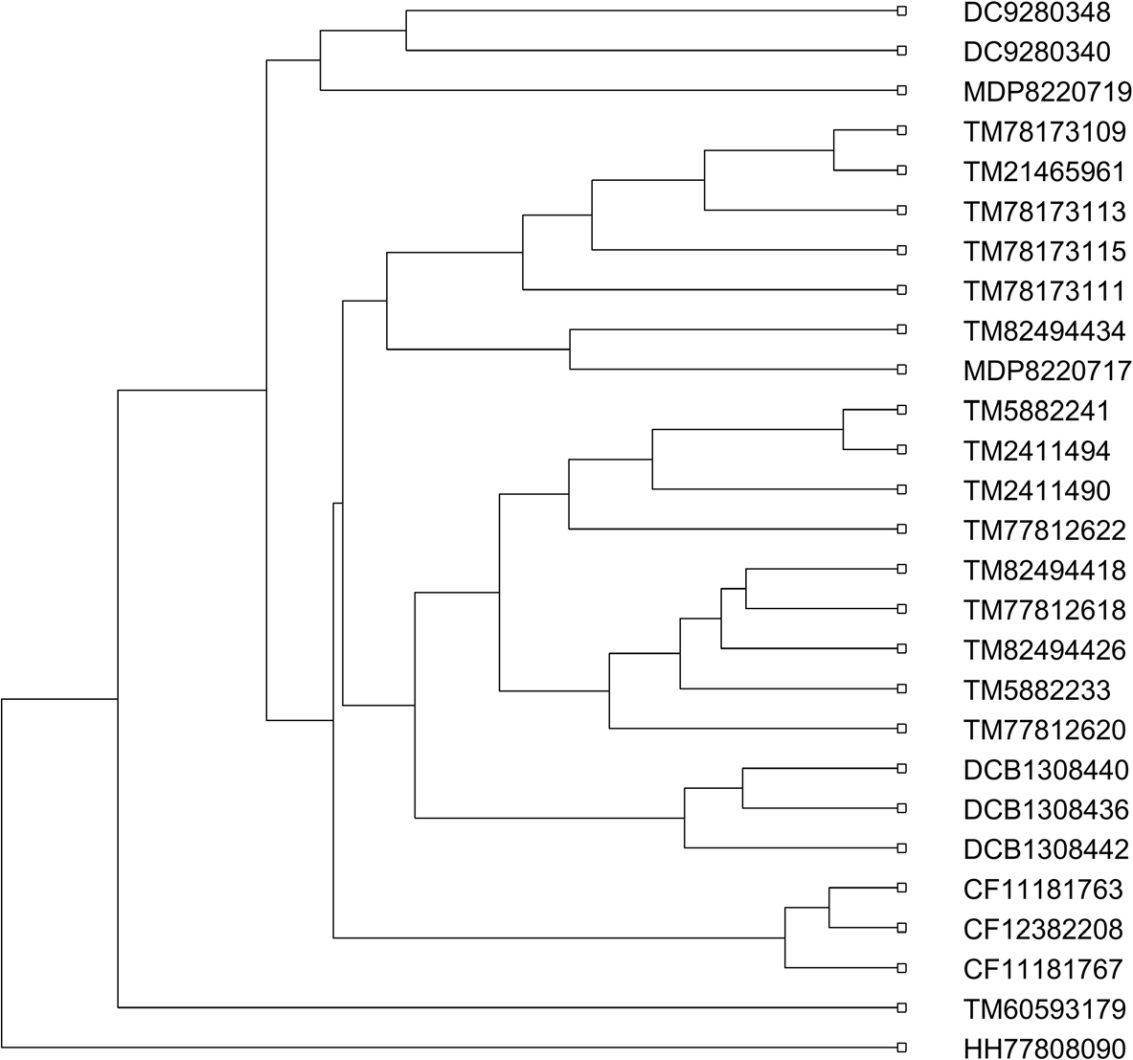
Phylogenetic tree of the 27 AFPs constructed without considering the distribution information of points

After comparison, we found that it performs much worse than DCGR, especially on the second and fifth data sets, whose phylogenetic trees are shown in Figs. 6 and 7, respectively. For the 36 proteins in Fig. 6, the FCGR method without considering the distribution information of points in CGR image separated none of the five protein families from the others, making the phylogenetic tree in quite a mess. For the 27 AFPs in Fig. 7, it erroneously clustered the TM proteins into three branches, and separated the 2 MD proteins in two branches. Similar results could be seen on the other three data sets (see Additional file 1: Figures S22-S24 for details). Therefore, it is easy to conclude that the distribution information of points in the CGR image shows great importance in the method design based on the CGR.

## Discussion

Feature extractions of protein sequences play an important role in protein sequence studies, e.g. the predictions of protein functions or protein-protein interactions. Although a great amount of methods have been proposed for extracting features of protein sequences, most of them showed great limits in practical applications. Many studies have showed that the CGR-based strategy would be one of the most useful approaches for protein feature extractions, and the so-called FCGR method is currently the most frequently used method based CGR, however a large amount of useful information, e.g. physicochemical properties of amino acids and the distribution information of points in the CGR image were not taken into consideration in the method design of FCGR.

In this paper, we proposed a new feature extraction method for protein sequences based on the CGR, where two novel techniques are developed in the design of the method DCGR. (1) During the construction of CGR curves, we designed a technique attempting to make full use of the physicochemical properties of amino acids, so the constructed CGR curves contain more useful information, making it more reliable. (2) In the conversion of the CGR curves into numerical characteristics, different from traditional FCGR methods, we opened a new door by integrating the distribution information of points in the CGR image into the method design of DCGR, which is proved quite important and makes the extracted features more efficient.

Compared with previously published methods including ClustalW on five most frequently used data sets, DCGR consistently performs the best. In addition, the method DCGR proposed in this paper could be used not only in the similarity analyses of protein sequences, but also in the areas of investigating protein classification or prediction problems in bioinformatics, which will be the topics in our future studies.

## Conclusions

We have developed a practically effective method for feature extractions of protein sequences. It is the first CGR-based method by effectively integrating the physicochemical properties of amino acids and the distribution information of points in the CGR image into the method design. Results show that DCGR is currently the most accurate method for protein feature extractions, and demonstrate great potentials for the studies of protein similarity analyses, protein function predictions and protein-protein interactions.

## Methods

### AAindex database

AAindex is a database of numerical indices representing various physicochemical and biochemical properties of amino acids and amino acid pairs [57, 58]. The latest version is the 9.2 release, which currently contains 566 indices. An amino acid index is a set of 20 numerical values representing any of the different physicochemical properties of the 20 amino acids. Here, we selected 158 indices for the following applications after removing all the redundant ones, in which different amino acids have the same value, and the 158 selected indices have been detailed in Additional file 1: Notes 1.1.

### Construction of CGR curves for protein sequences

As did previously, the 4-CGR, 5-CGR or 6-CGR using only 4, 5 or 6 letters to represent protein sequences would result in loss of sequence information, since the amino acids belonging to the same group are considered identical. In order to avoid the loss of sequence information, we developed the DCGR based on 20-CGR mentioned above, where it is a highly challenging task to reasonably locate the 20 amino acids at equal distances on the circumference of a unit circle, as there are up to 20! possible arrangements. In this study, we first developed a novel technique specially to solve the problem of amino acid arrangement by applying the physicochemical properties selected from AAindex database. Then the CGR curves of a protein sequence could be constructed according to the arrangements of the 20 amino acids on the unit circle.

#### Arranging the 20 amino acids on the circumference of a unit circle

In order to fully use the physicochemical properties of the amino acids, we first sort the 20 amino acids according to their physicochemical indices in ascending order. Then the 20 amino acids are arranged in order on the circumference of a unit circle by the following equation,

1 $$ \varphi \left({X}_i\right)=\left(\cos \frac{2\pi i}{20},\sin \frac{2\pi i}{20}\right),i=1,2,\cdots, 20 $$

where *X*_*i*_ represents each of the 20 amino acids.

#### Building CGR curves for protein sequences

Given a protein sequence *S* with *N* amino acids *S = s*_1_ *s*_2…_*s*_N_, the CGR curve is constructed by successively connecting *N* points corresponding to the *N* amino acids, the coordinate of which are determined as follows. The first point is specified as the midpoint of the center of the unit circle and the point of the circumference corresponding to the first amino acid *s*_1_. For the *i*-th amino acid *s*_*i*_, its point coordinate is defined as the midpoint of the (*i-*1)-th point and the point of the circumference corresponding to the amino acid *s*_i_. In detail, the iterative procedure can be formulated as:

2 $$ \psi \left({s}_i\right)=\frac{1}{2}\left(\psi \left({s}_{i-1}\right)+\varphi \left({s}_i\right)\right),i=1,2,\cdots, N $$

where *ψ*(*s*_*i*_) represents the coordinate of the point corresponding to the *i*-th amino acid *s*_*i*_, and *ψ*(*s*_*0*_) is set to be (0, 0).

Corresponding to each of the 158 selected physicochemical properties, we can obtain an exclusive arrangement of 20 amino acids on the circumference of a unit circle, and then a CGR curve for a protein sequence. Thus, 158 intrinsically different CGR curves could be constructed for each protein sequence corresponding to the 158 physicochemical properties of amino acids.

### Conversion of CGR curves into numerical characteristics

After obtaining 158 CGR curves for each protein sequence, another challenging task is to effectively convert the CGR curves into numerical characteristics, which could then be used for similarity analysis among protein sequences. In this study, we developed a new method for extracting numerical characteristics from CGR curves as follows.

Given a protein sequence *S*, we can obtain 158 different CGR curves falling in a unit circle. In order to extract features from protein sequence, for each of the 158 CGR curves, we first split the unit circle into four segments according to the four quadrants. Then, we compute pairwise distances between points in each segment and obtain four distance matrices for a CGR curve. By computing their leading eigenvalues, we obtain a 4-dimensional vector which is taken as the numerical characteristics of the CGR curve. All of the numerical characteristics of 158 CGR curves are integrated into a 632-dimensional vector which is taken as the feature vector of the protein sequence.

Given a data set consisting of *N* protein sequences, we can obtain an *N* × 632 feature matrix, each row of which corresponds to a feature vector of a protein sequence. Since the dimension of the feature vectors is very high, there may be redundancies and noises in them. We use the Principal Component Analysis (PCA) to reduce the dimensionality of the feature vectors. The reduced feature vectors are then applied to analyze the similarity of protein sequences.

## Additional file

Additional file 1: This file contains supplementary notes, figures and tables. (PDF 1238 kb)

## Data Availability

See Additional file 1 for the availability of the tested protein sequences, and DCGR is a free, open-source package available from https://sourceforge.net/projects/transcriptomeassembly/files/Feature%20Extraction.

## Abbreviation

CGR: Chaos Game Representation
