# Supplementary material for: DCGR: feature extractions from protein sequences based on CGR via remodeling multiple information
Source: BMC Bioinformatics. 2019 Jun 20;20:351. doi: 10.1186/s12859-019-2943-x (PMC6587251; doi:10.1186/s12859-019-2943-x)
Supplement: Supplementary file 1 — This file contains supplementary notes, figures and tables. (PDF 1238 kb) [file 12859_2019_2943_MOESM1_ESM.pdf]

1. Supplementary Notes

1.1 Selected 158 physicochemical properties

1) Signal sequence helical potential
2) Membrane-buried preference parameters
3) Average flexibility indices
4) Information value for accessibility; average fraction 23%
5) Retention coefficient in TFA
6) alpha-NH chemical shifts
7) alpha-CH chemical shifts
8) Normalized frequency of extended structure
9) A parameter defined from the residuals obtained from the best correlation of the Chou-Fasman parameter of beta-sheet
10) Average volume of buried residue
11) Frequency of the 2nd residue in turn
12) Frequency of the 4th residue in turn
13) Normalized frequency of the 2nd and 3rd residues in turn
14) Normalized hydrophobicity scales for alpha-proteins
15) Membrane preference for cytochrome b: MPH89
16) Average membrane preference: AMP07
17) Consensus normalized hydrophobicity scale
18) Hydrophobic parameter pi
19) Aperiodic indices
20) Aperiodic indices for beta-proteins
21) Aperiodic indices for alpha/beta-proteins
22) Partition energy
23) Heat capacity
24) Absolute entropy
25) Entropy of formation
26) Normalized relative frequency of alpha-helix
27) Average accessible surface area
28) Sequence frequency
29) Average relative probability of helix
30) Average relative probability of inner helix
31) Flexibility parameter for one rigid neighbor
32) Side chain interaction parameter
33) Side chain interaction parameter
34) Distance between C-alpha and centroid of side chain
35) Radius of gyration of side chain

36) Normalized frequency of beta-sheet
37) Normalized frequency of reverse turn
38) Average surrounding hydrophobicity
39) Normalized frequency of extended structure
40) Refractivity
41) Retention coefficient in HPLC
42) Retention coefficient in NaClO4
43) Effective partition energy
44) AA composition of total proteins
45) Normalized composition from animal
46) AA composition of mt-proteins from fungi and plant
47) AA composition of membrane proteins
48) Transmembrane regions of non-mt-proteins
49) Transmembrane regions of mt-proteins
50) AA composition of CYT of single-spanning proteins
51) AA composition of CYT2 of single-spanning proteins
52) AA composition of EXT of single-spanning proteins
53) AA composition of EXT2 of single-spanning proteins
54) AA composition of MEM of single-spanning proteins
55) AA composition of CYT of multi-spanning proteins
56) AA composition of EXT of multi-spanning proteins
57) 8 A contact number
58) 14 A contact number
59) Average non-bonded energy per atom
60) Long range non-bonded energy per atom
61) Average non-bonded energy per residue
62) Short and medium range non-bonded energy per residue
63) Optimized beta-structure-coil equilibrium constant
64) Optimized propensity to form reverse turn
65) Optimized transfer energy parameter
66) Optimized average non-bonded energy per atom
67) Optimized side chain interaction parameter
68) Normalized frequency of alpha-helix from CF
69) Normalized frequency of alpha-helix in alpha+beta class
70) Average gain in surrounding hydrophobicity
71) Surrounding hydrophobicity in alpha-helix
72) Average number of surrounding residues
73) Hydrophobicity
74) Relative frequency in reverse-turn
75) Weights for alpha-helix at the window position of -3

76) Weights for alpha-helix at the window position of 0
77) Weights for alpha-helix at the window position of 3
78) Weights for beta-sheet at the window position of 0
79) Weights for coil at the window position of -1
80) Weights for coil at the window position of 0
81) Weights for coil at the window position of 1
82) Weights for coil at the window position of 5
83) Average reduced distance for side chain
84) Average relative fractional occurrence in AR (i)
85) Average relative fractional occurrence in A0 (i-1)
86) Value of theta (i)
87) Transfer free energy from vap to chx
88) Accessible surface area
89) Information measure for middle helix
90) Mean area buried on transfer
91) Principal component I
92) Principal component III
93) Principal component IV
94) Normalized frequency of isolated helix
95) Normalized frequency of extended structure
96) Normalized frequency of chain reversal R
97) Normalized frequency of coil
98) Relative population of conformational state A
99) Relative population of conformational state C
100) Transfer free energy to lipophilic phase
101) Average interactions per side chain atom
102) Hydration potential
103) Principal property value z1
104) Principal property value z2
105) Principal property value z3
106) Activation Gibbs energy of unfolding
107) Normalized positional residue frequency at helix termini C3
108) Normalized flexibility parameters
109) Free energy in alpha-helical region
110) Free energy in beta-strand conformation
111) p-Values of thermophilic proteins based on the distributions of B values
112) Distribution of amino acid residues in the 18 non-redundant families of thermophilic proteins
113) Distribution of amino acid residues in the alpha-helices in thermophilic mesophilic proteins
114) Hydropathy scale based on self-information values in the two-state model (16% accessibility)
115) Hydropathy scale based on self-information values in the two-state model (25% accessibility)

116) Hydropathy scale based on self-information values in the two-state model (50% accessibility)
117) Alpha-helix propensity derived from designed sequences
118) Surface composition of amino acids in intracellular proteins of thermophiles
119) Surface composition of amino acids in intracellular proteins of mesophiles
120) Surface composition of amino acids in nuclear proteins
121) Interior composition of amino acids in intracellular proteins of thermophiles
122) Interior composition of amino acids in intracellular proteins of mesophiles
123) Interior composition of amino acids in extracellular proteins of mesophiles
124) Entire chain composition of amino acids in intracellular proteins of thermophiles
125) Entire chain composition of amino acids in extracellular proteins of mesophiles
126) Entire chain compositino of amino acids in nuclear proteins
127) Volumes including the crystallographic waters using the ProtOr
128) Volumes not including the crystallographic waters using the ProtOr
129) Hydrophobicity scales
130) Hydrophobicity coefficient in RP-HPLC
131) Hydrophobicity coefficient in RP-HPLC
132) Interactivity scale obtained from the contact matrix
133) Interactivity scale obtained by maximizing the mean of correlation coefficient over single-domain globular proteins
134) Interactivity scale obtained by maximizing the mean of correlation coefficient over pairs of sequences sharing the TIM barrel fold
135) Linker propensity index
136) Linker propensity from 2-linker dataset
137) Linker propensity from medium dataset
138) Linker propensity from long dataset
139) Linker propensity from helical (annotated by DSSP) dataset
140) Linker propensity from non-helical (annotated by DSSP) dataset
141) Linker index
142) Mean volumes of residues buried in protein interiors
143) Average volumes of residues
144) Hydrostatic pressure asymmetry index
145) Average internal preferences
146) Apparent partition energies calculated from Janin index
147) Apparent partition energies calculated from Chothia index
148) Weights from the IFH scale
149) Hydrophobicity index
150) NNEIG index
151) SWEIG index
152) PRIFT index
153) ALTFT index

154) ALTLS index
155) TOTFT index
156) TOTLS index
157) Relative partition energies derived by the Bethe approximation
158) Hydrophobicity index

1.2 Accession numbers of the 50 beta-globin proteins

Human (AAA16334.1), Pigeon (P11342.1), Goshawk (P08851.1), Black bear (P68012.1)), Lesser panda (P18982.1), Asiatic elephant (P02084.1), Giant panda (P18983.2), African elephant (P02085.1), Sheep (P02075.2), Tortoise (P83123.3), Duck (P02114.2), Grivet (P02028.1), Mallard (P02115.1), Gorilla (P02024.2), Goose (P02117.1), Shark (P02143.1), Rat (CAA33114.1), Hippopotamus (P19016.1), Penguin (P80216.1), Horse (P02062.1), Swift (P15165.1), Gibbon (P02025.1), Coyote (P60525.1), Whale (P18984.1), Catfish (O13163.2), Bat (P24660.1), Bison (P09422.1), Red fox (P21201.1), Swan (P68945.1), Marmot (P08853.1), Buffalo (P67820.1), Salmon (Q91473.3), Dog (P60524.1), Sparrow (P07406.1), Chimpanzee (P68873.2), Pheasant (P02113.1), Dolphin (P18990.1), Flamingo (P02121.1), Goldfish (P02140.1), Pig (P02067.3), Polar bear (P68011.1), Dragonfish (ADD73488.1), Rhinoceros (P09907.1), Parakeet (P21668.1), Chicken (P02112.2), Zebra (P67824.1), Wolf (P60526.1), Cod (O13077.2), Turtle (P13274.1), and Langur (P02032.1)

2. Supplementary Tables

Table S1. The information for nine ND5 protein sequences

No.	Species	ID(NCBI)	length
1	Human (Homo sapiens)	AP 000649	603
2	Gorilla (Gorilla gorilla)	NP 008222	603
3	Common chimpanzee (Pan troglodytes)	NP 008196	603
4	Pigmy chimpanzee (Pan paniscus)	NP 008209	603
5	Fin whale (Balenoptera physalus)	NP 006899	606
6	Blue whale (Balenoptera musculus)	NP 007066	606
7	Rat (Rattus norvegicus)	AP 004902	610
8	Mouse (Mus musculus)	NP 904338	607
9	Opossum (Didelphis virginiana)	NP 007105	602

Table S2. The distance matrix of the nine ND5 protein sequences calculated by our method

	Human	Gorilla	C.chim	P.chim	F.whale	B.whale	Rat	Mouse	Opossum
Human	0	89	45	70	125	125	183	202	233
Gorilla		0	82	82	99	109	165	182	200
C.chim			0	29	121	119	172	191	226

P.chim	0	111	108	156	178	206
F.whale		0	20	145	160	173
B.whale			0	138	153	178
Rat				0	109	143
Mouse					0	173
Opossum						0

Table S3. The information for transferrin sequences from 25 vertebrates

Name	Species	Accession No.	Length
Human TF	Homo sapiens	S95936	698
Rabbit TF	Oryctolagus coniculus	X58533	695
Rat TF	Rattus norvegicus	D38380	698
Cow TF	Bos Taurus	U02564	704
Buffalo LF	Bubahts arnee	AJ005203	708
Cow LF	Bos Taurus	X57084	708
Goat LF	Copra hircus	X78902	708
Camel LF	Camelus dromedarius	AJ131674	708
Pig LF	Sus scrofa	M92089	704
Human LF	Homo sapiens	NM_002343	710
Mouse LF	Mus musculus	NM_008522	707
Possum TF	Trichosurus vulpecula	AF092510	711
Frog TF	Xenopus laevis	X54530	702
Medaka TF	Oryzias latipes	D64033	690
Japanese flounder TF	Paralichthys olivaceus	D88801	685
Atlantic salmon TF	Salmo salar	L20313	690
Brown trout TF	Salmo trutta	D89091	691
Lake trout TF	Salvelinus namaycush	D89090	691
Brook trout TF	Salvelinus fontinalis	D89089	691
Japanese char TF	Salvelinus pluvius	D89088	691
Chinook salmon TF	Oncorhynchus tshawytscha	AH008271	677
Coho salmon TF	Oncorhynchus hisutch	D89084	691
Sockeye salmon TF	Oncorhynchus nerka	D89085	691
Rainbow trout TF	Oncorhynchus mykiss	D89083	691
Amago salmon TF	Oncorhynchus masou	D89086	691

Table S4. The information for 27 antifreeze proteins

Sequence name	Accession	Species
DC9280348	AAF86363.1	Dendroides Canadensis
DC9280340	AAF86359.1	Dendroides canadensis
TM82494434	ABB79834.1	Tenebrio molitor
TM82494426	ABB79830.1	Tenebrio molitor
TM82494418	ABB79826.1	Tenebrio molitor
TM2411490	AAB70750.1	Tenebrio molitor
TM60593179	AAX28872.1	Tenebrio molitor
TM5882241	AAD55260.1	Tenebrio molitor
TM5882233	AAD55256.1	Tenebrio molitor
TM78173115	ABB29474.1	Tenebrio molitor
TM78173113	ABB29473.1	Tenebrio molitor
TM78173111	ABB29472.1	Tenebrio molitor
TM78173109	ABB29471.1	Tenebrio molitor
TM77812622	ABB03885.1	Tenebrio molitor
TM77812618	ABB03883.1	Tenebrio molitor
TM77812620	ABB03884.1	Tenebrio molitor
TM2411494	AAB70752.1	Tenebrio molitor
TM21465961	1L1IA	Tenebrio Molitor
MDP8220717	AAW67979.1	Microdera dzhungarica punctipennis
MDP8220719	AAW67980.1	Microdera dzhungarica punctipennis
DCB1308442	BAF43605.1	Dorcus curvidens binodulosus
DCB1308440	BAF43604.1	Dorcus curvidens binodulosus
DCB1308436	BAF43602.1	Dorcus curvidens binodulosus
CF11181767	AAF86612.1	Choristoneura fumiferana
CF11181763	AAF86610.1	Choristoneura fumiferana
CF12382208	ABI17375.1	Choristoneura fumiferana
HH77808090	ABB03725.1	Hypogastrura harveyi

3. Supplementary Figures

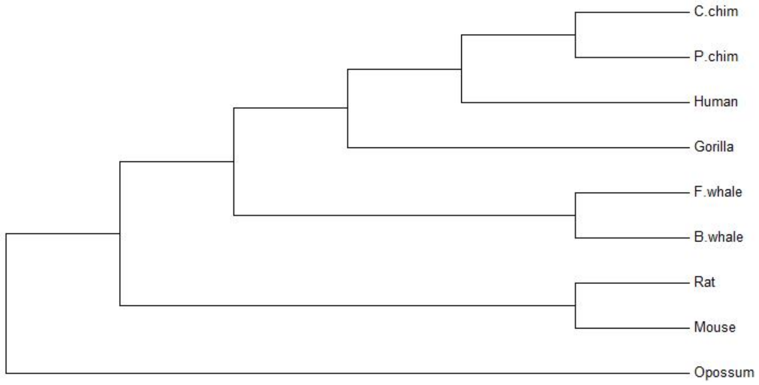

Figure S1. Phylogenetic tree of the nine ND5 proteins constructed by ClustalW.

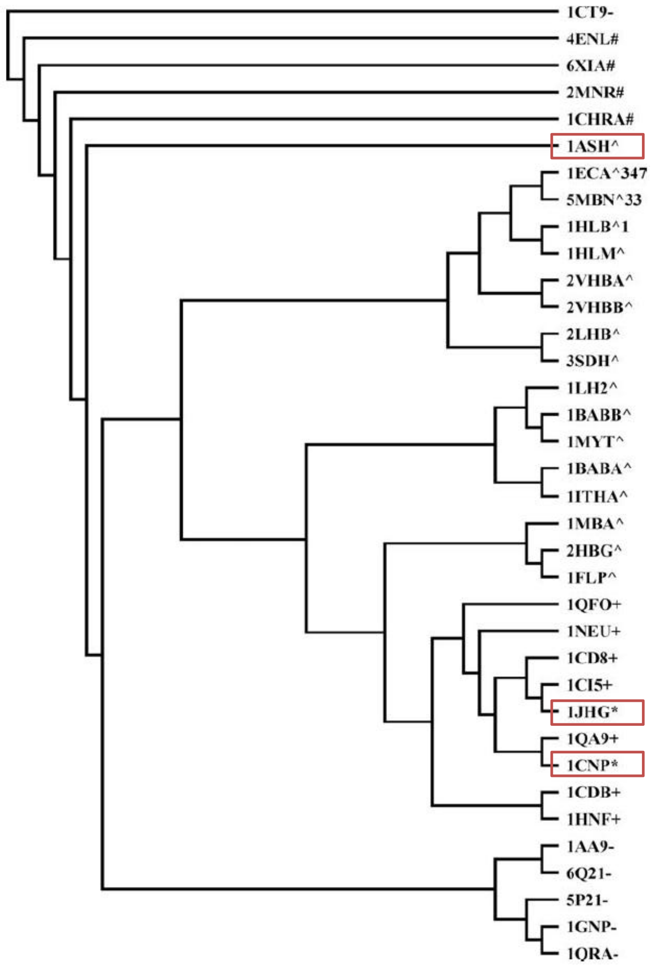

Figure S2. The phylogenetic tree of 36 protein sequences constructed in [20].

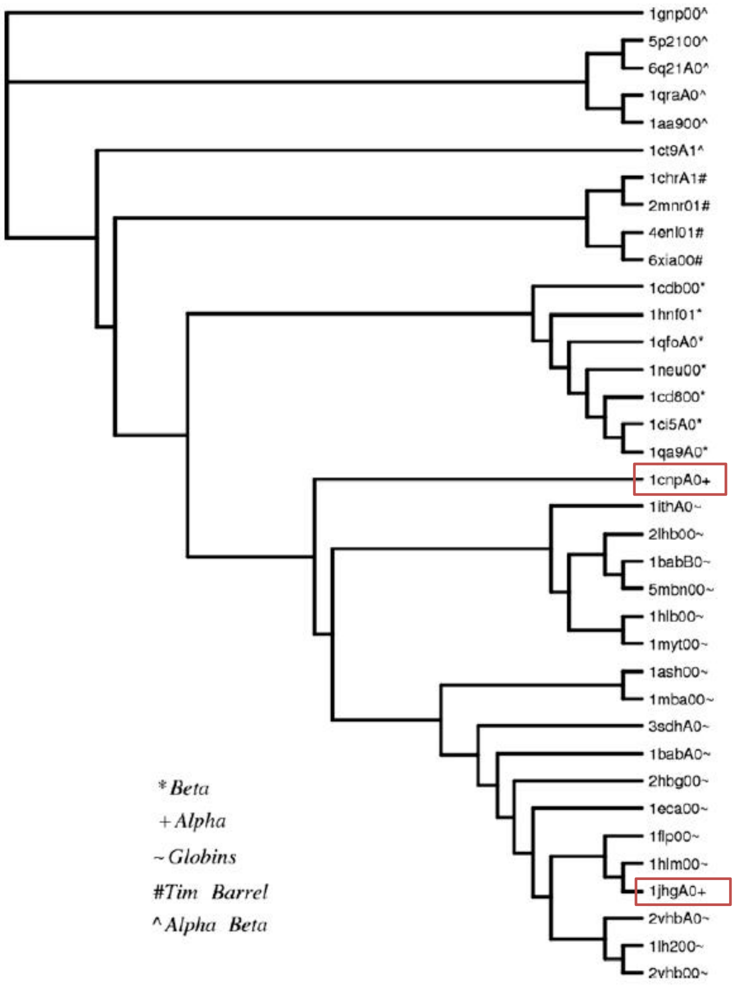

Figure S3. The phylogenetic tree of 36 protein sequences constructed in [45].

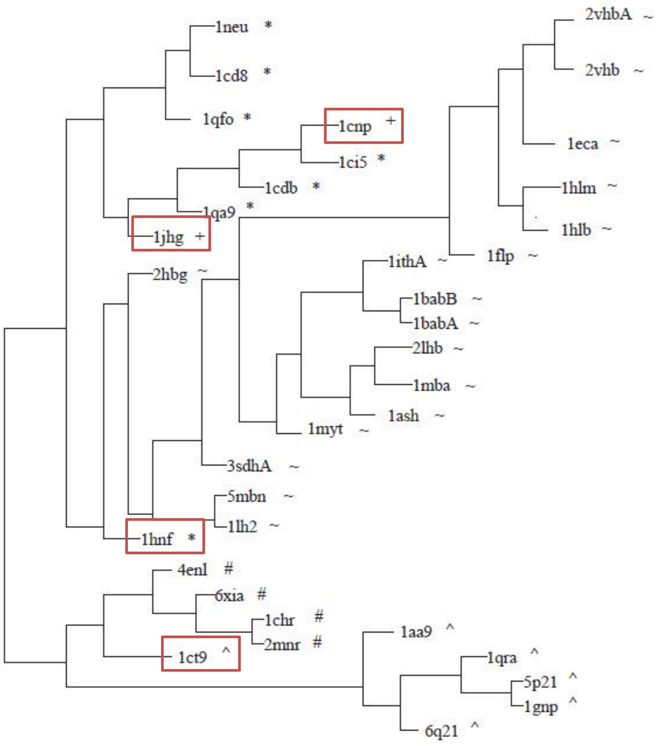

Figure S4. The phylogenetic tree of 36 protein sequences constructed in [46].

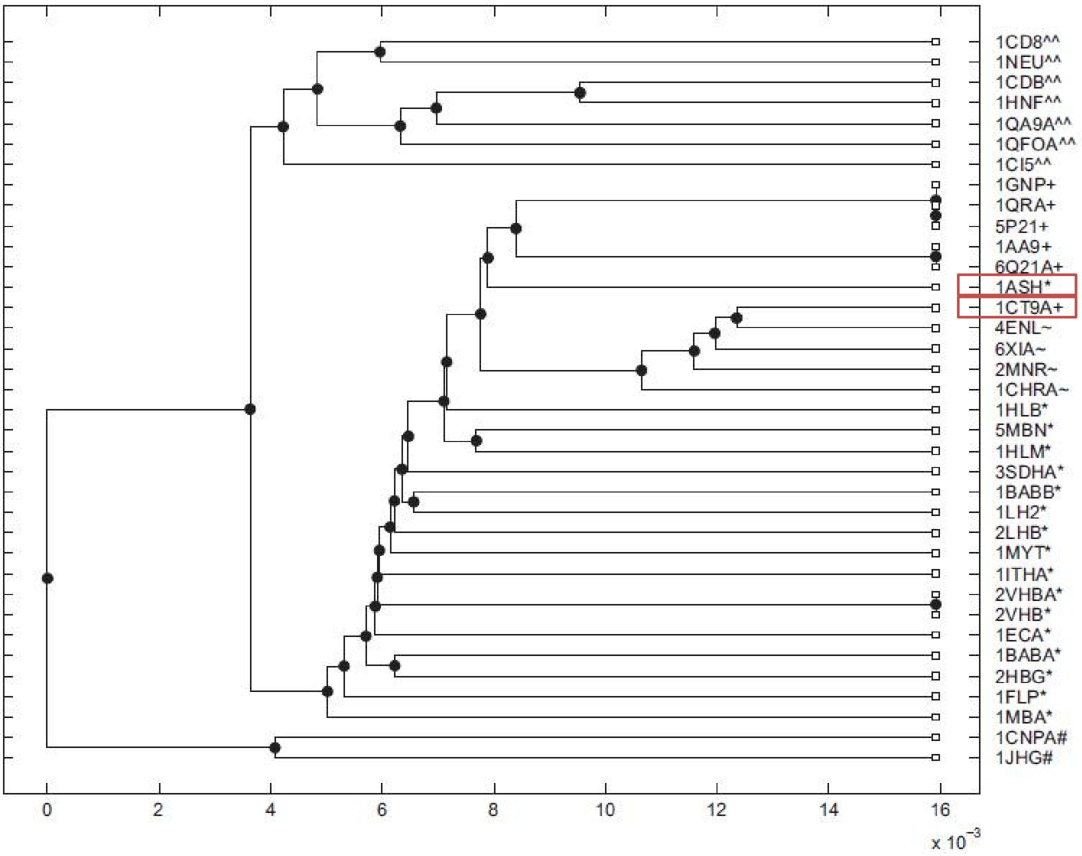

Figure S5. The phylogenetic tree of 36 protein sequences constructed in [47].

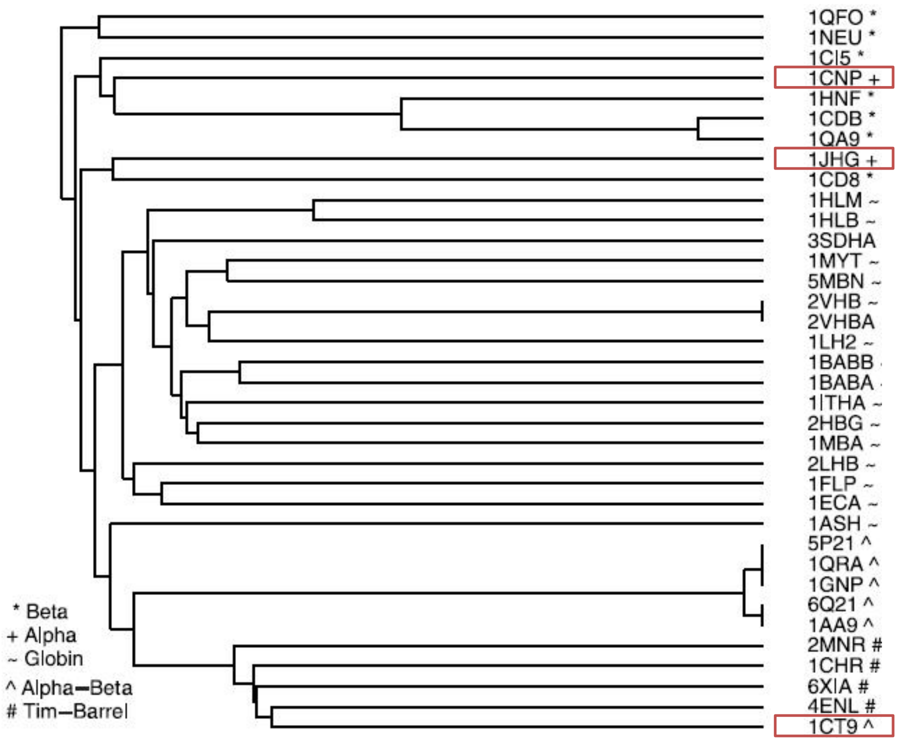

Figure S6. The phylogenetic tree of 36 protein sequences constructed in [48].

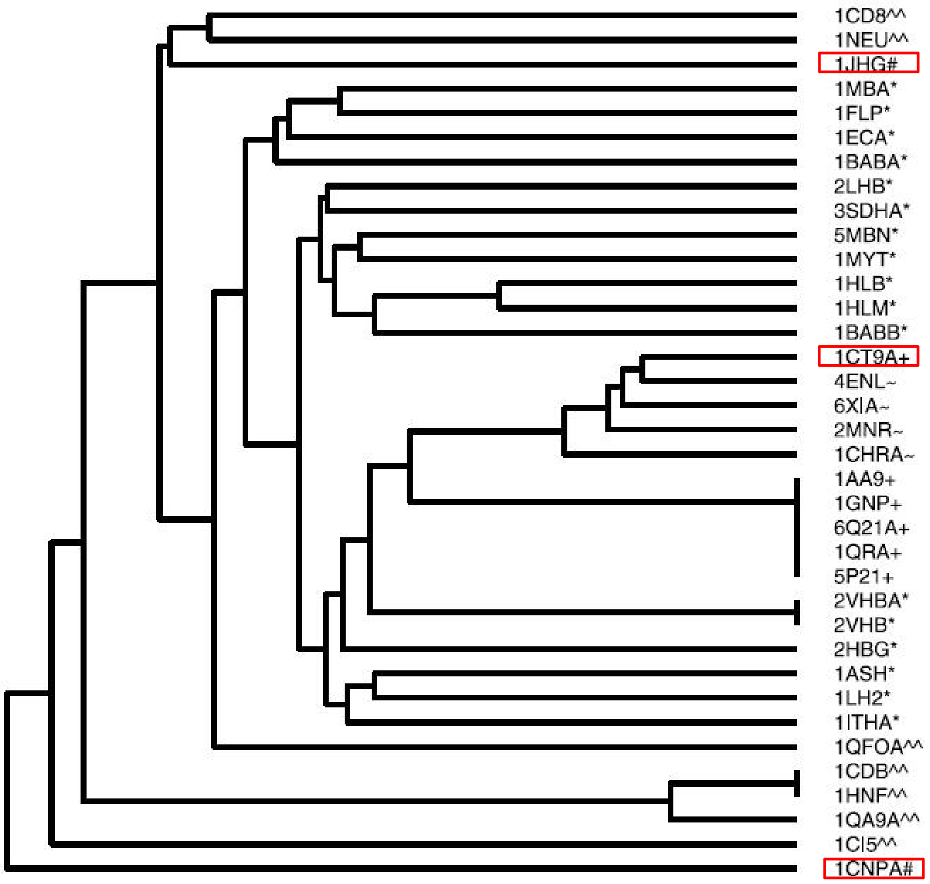

Figure S7. The phylogenetic tree of 36 protein sequences constructed in [57].

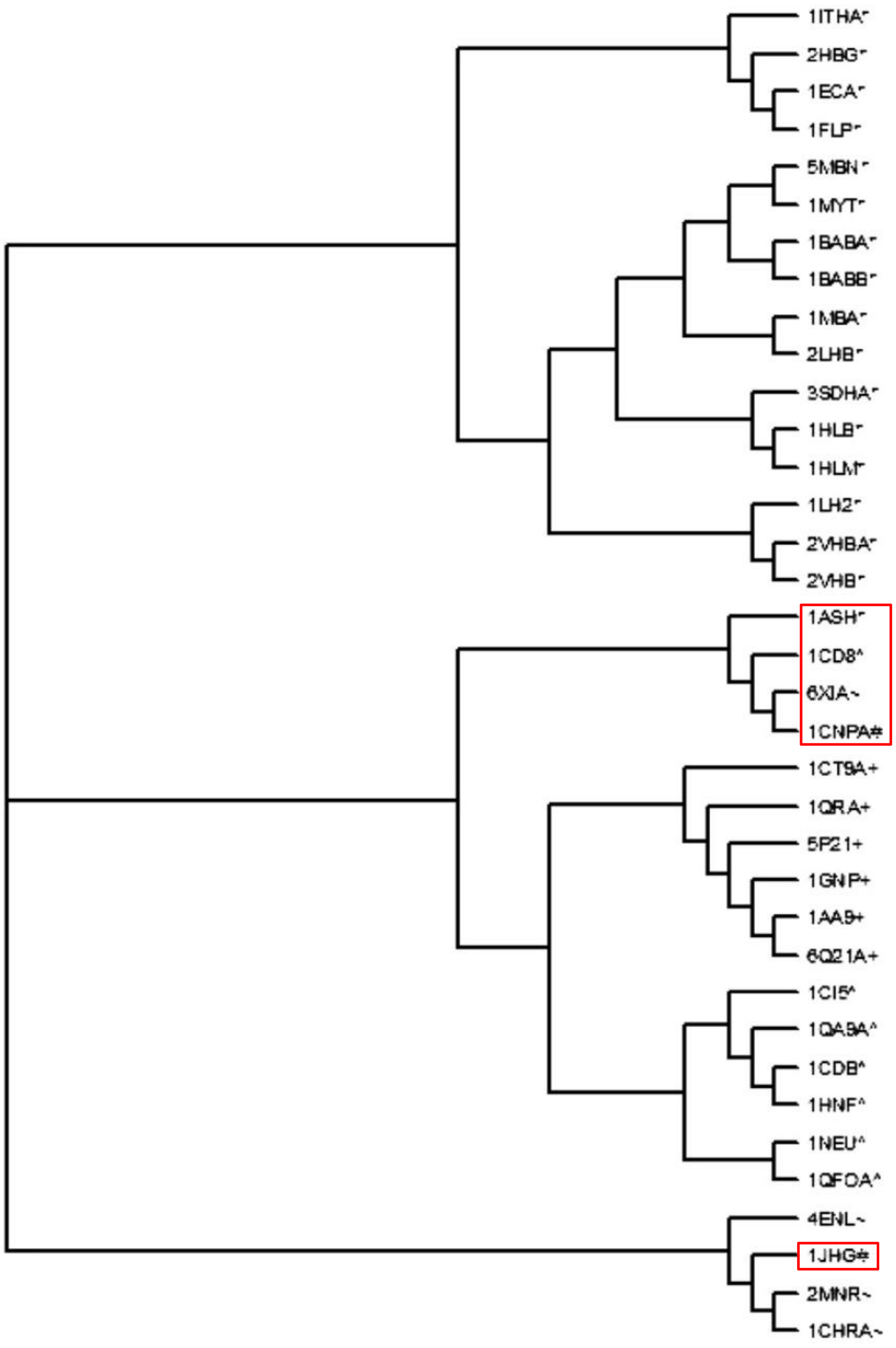

Figure S8. The phylogenetic tree of 36 protein sequences constructed by ClustalW in [57].

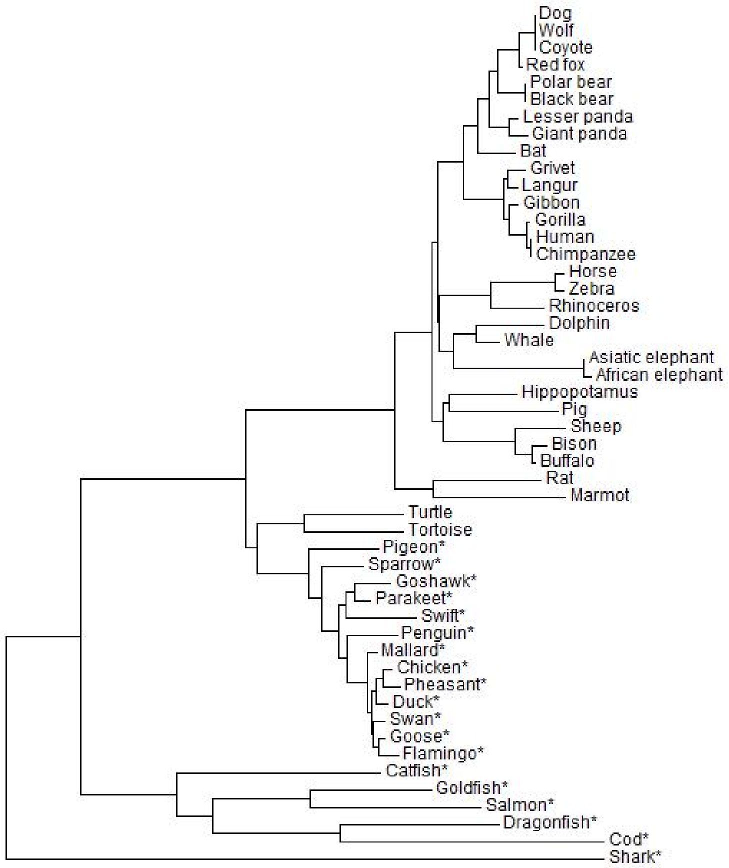

Figure S9. Phylogenetic tree of the 50 beta-globin protein sequences constructed by ClustalW

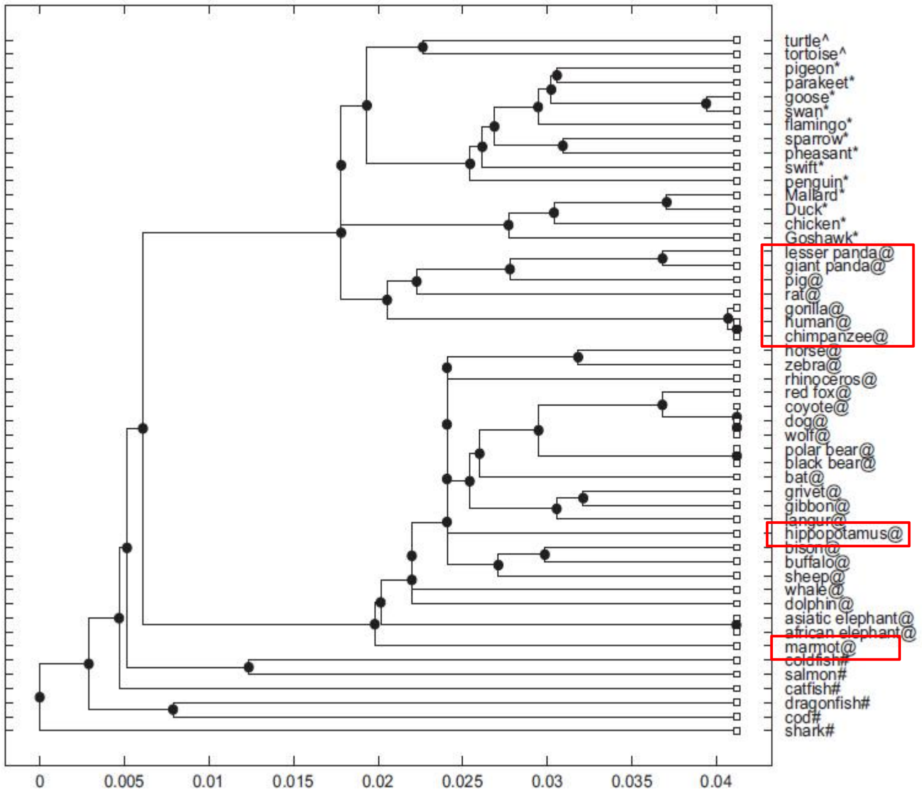

Figure S10. The phylogenetic tree of 50 beta-globin protein sequences constructed in [47].

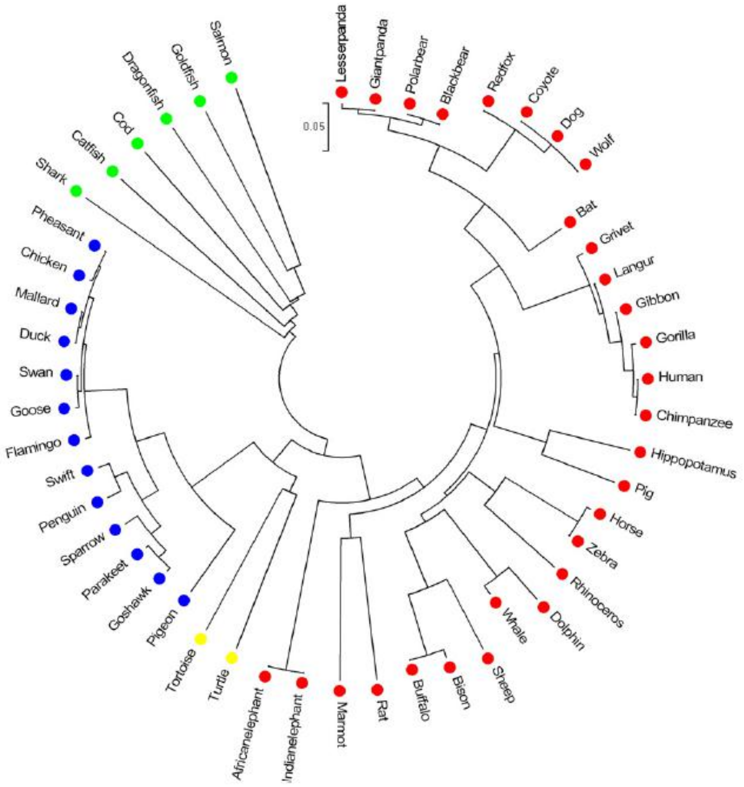

Figure S11. The phylogenetic tree of 50 beta-globin protein sequences constructed in [50].

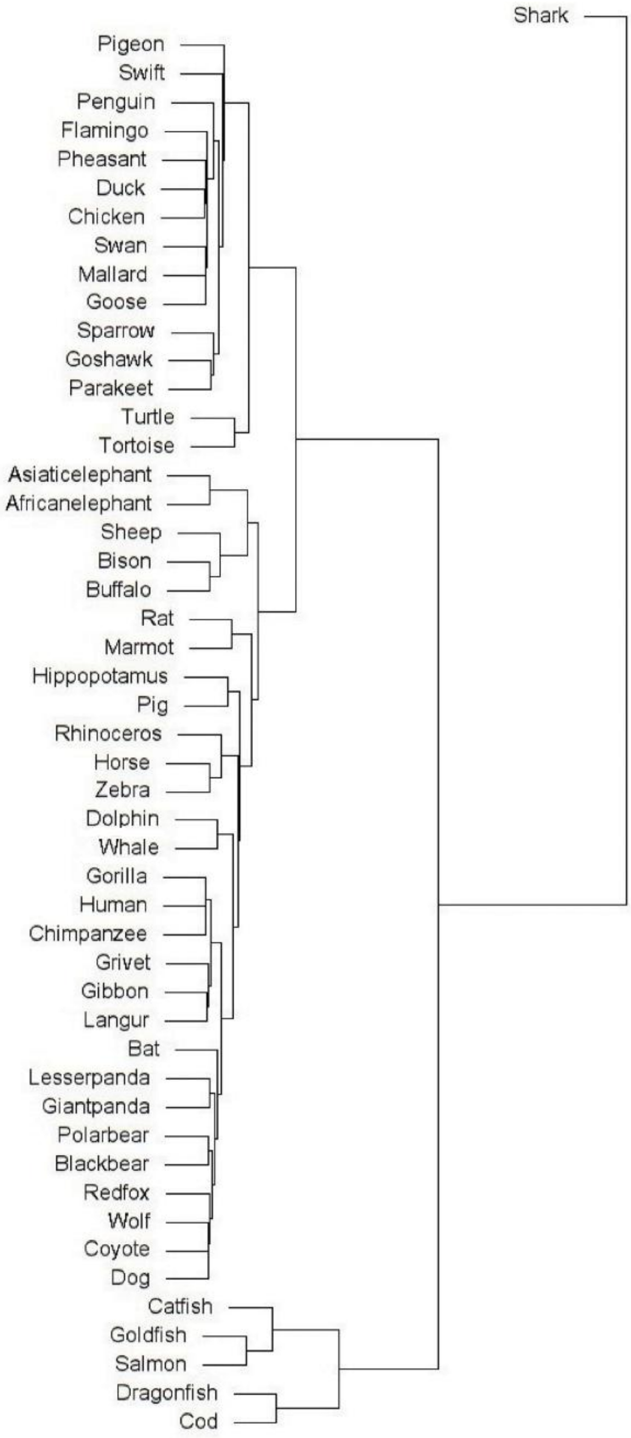

Figure S12. The phylogenetic tree of 50 beta-globin protein sequences constructed in [51].

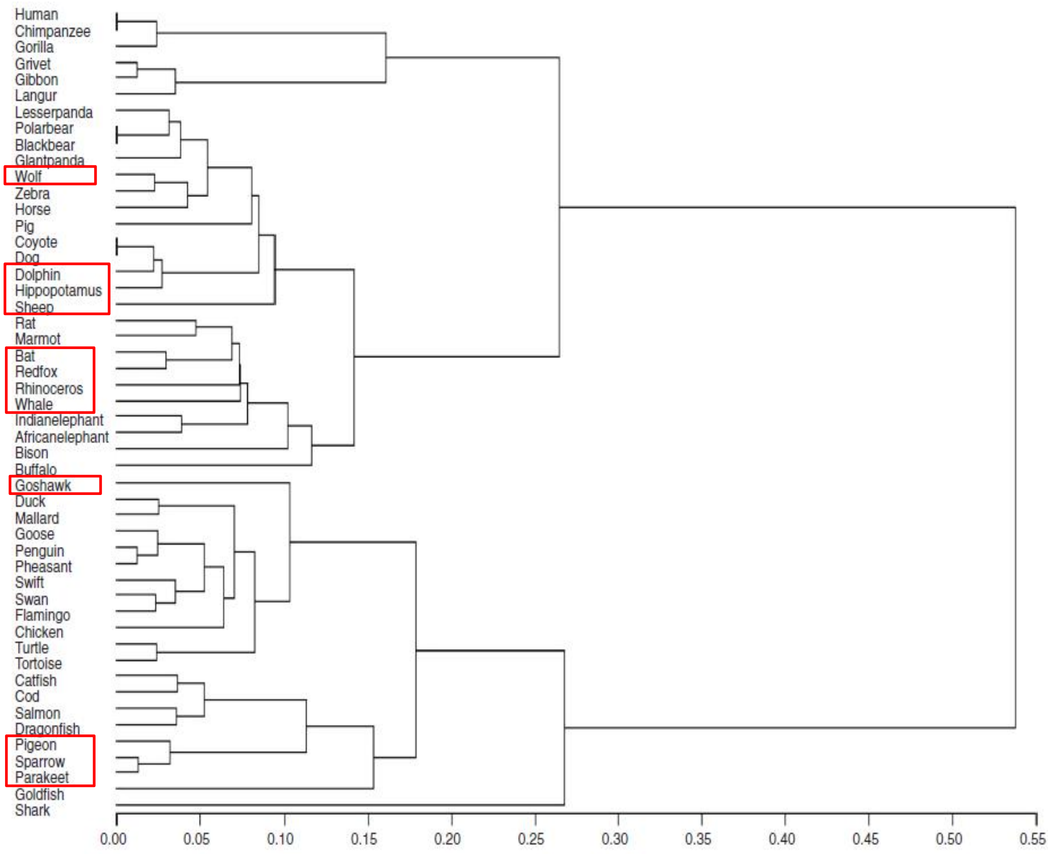

Figure S13. The phylogenetic tree of 50 beta-globin protein sequences constructed in [52].

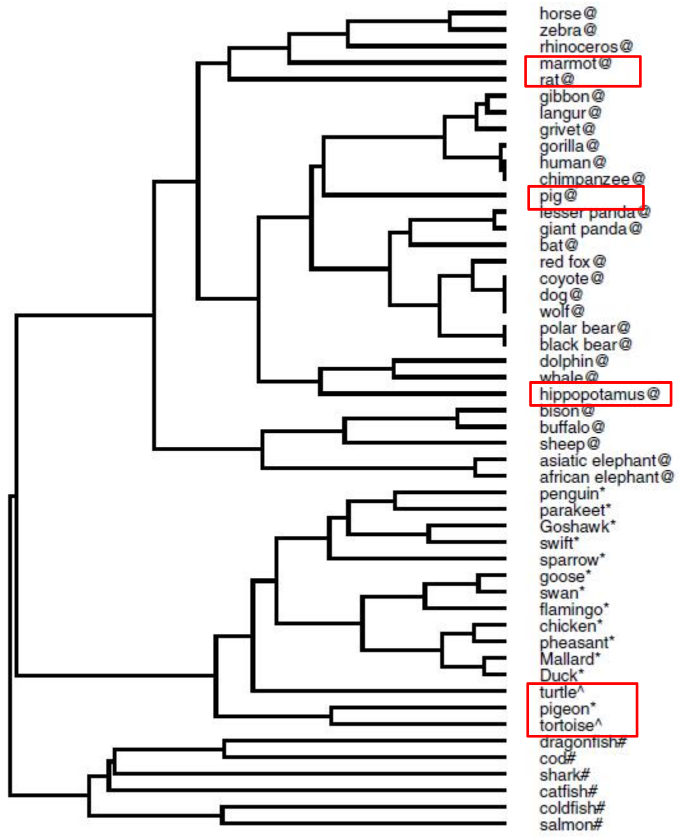

Figure S14. The phylogenetic tree of 50 beta-globin protein sequences constructed in [53].

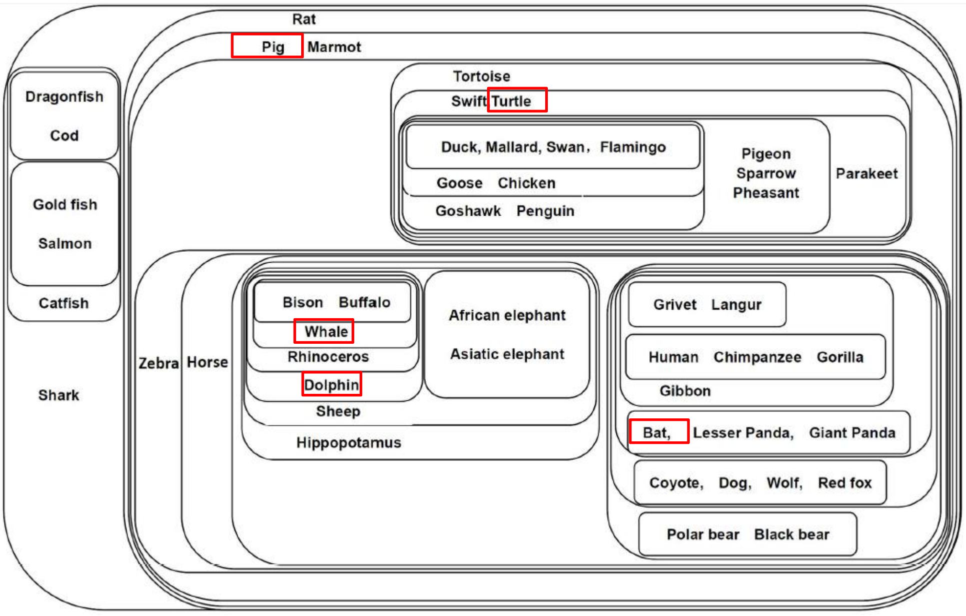

Figure S15. Classification results of 50 beta-globin protein sequences obtained in [54].

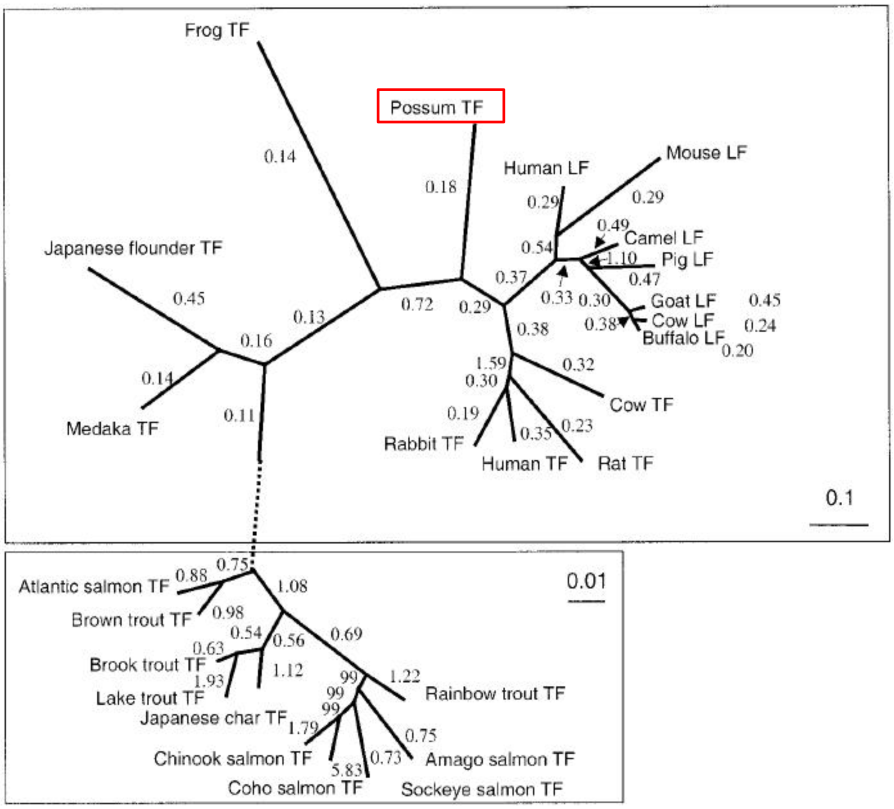

Figure S16. Phylogenetic tree of the 25 TFs constructed in [55]

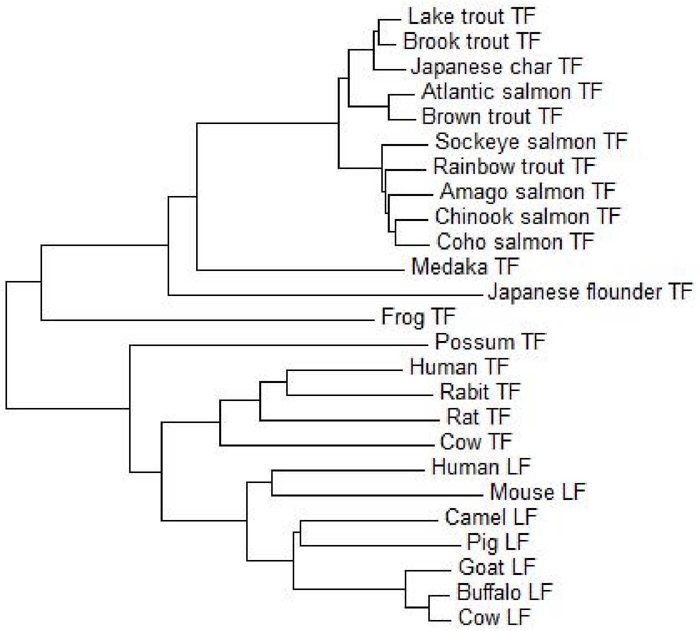

Figure S17. Phylogenetic tree of the 25 TFs constructed by ClustalW

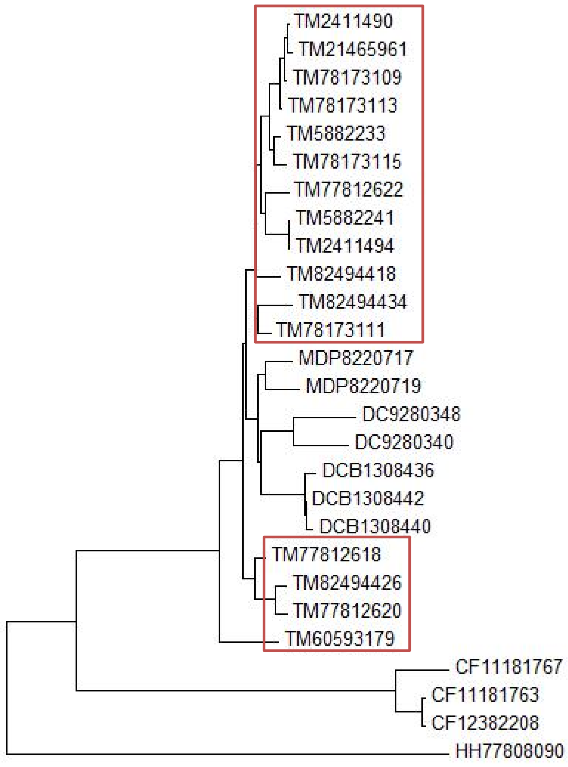

Figure S18. Phylogenetic tree of the 27AFPs constructed by ClustalW

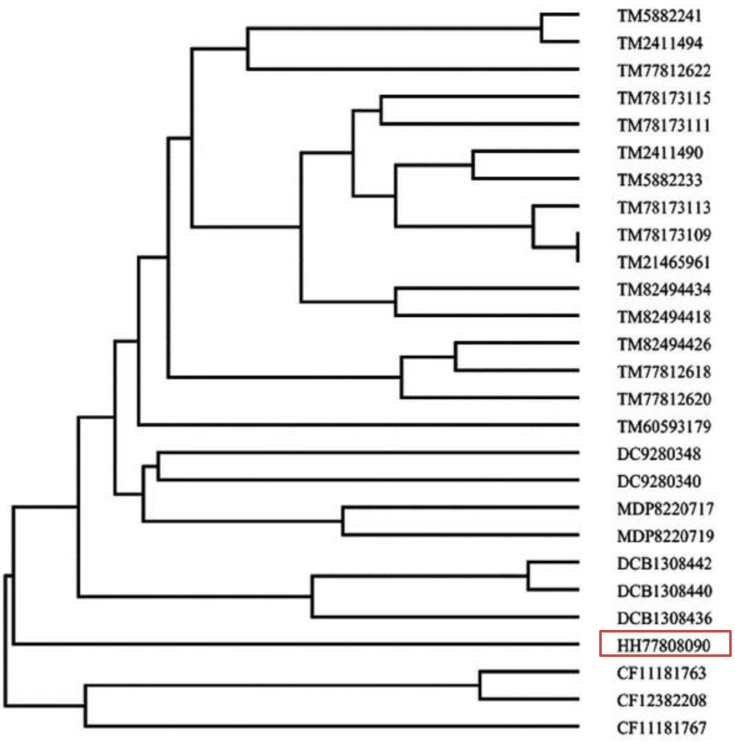

Figure S19. Phylogenetic tree of the 27 AFPs constructed in [53]

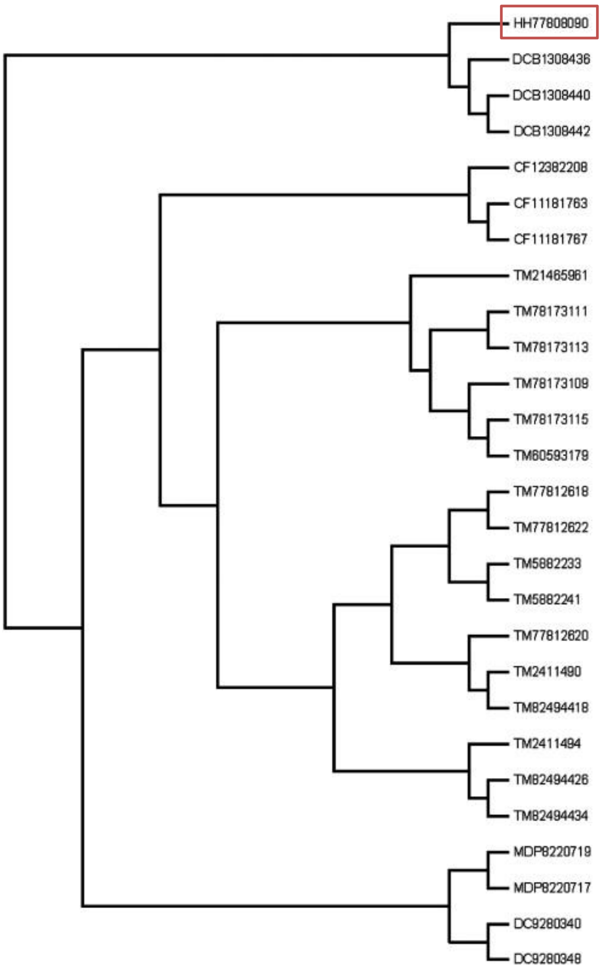

Figure S20. Phylogenetic tree of the 27 AFPs constructed in [56]

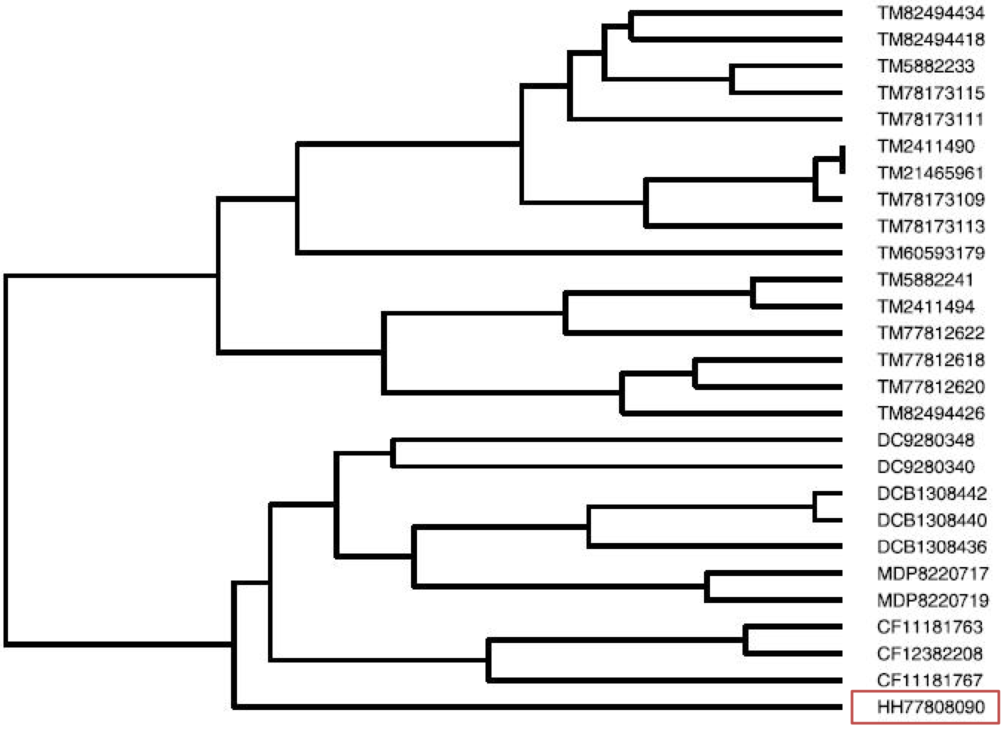

Figure S21. Phylogenetic tree of the 27 AFPs constructed in [57]

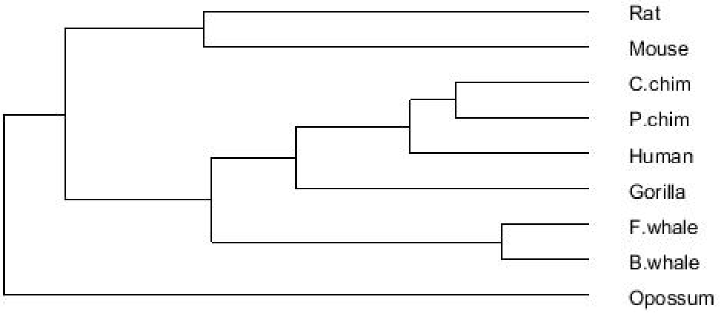

Figure S22. Phylogenetic tree of the nine ND5 proteins constructed by our method without considering the point distribution information.

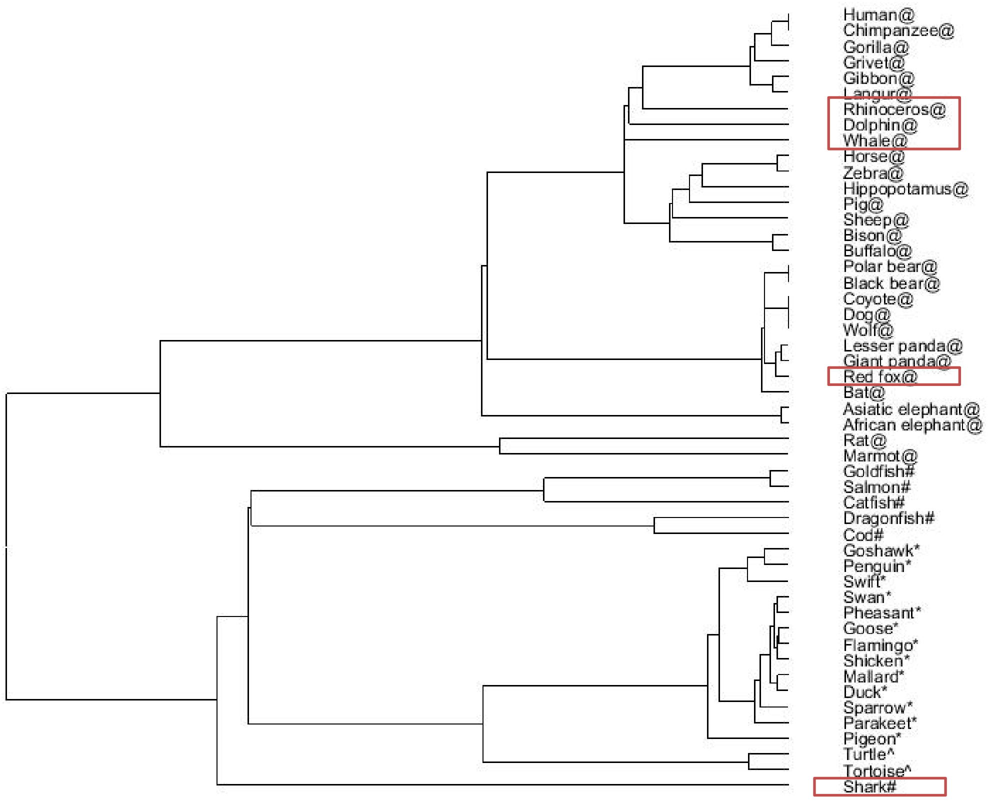

Figure S23. Phylogenetic tree of the 50 beta-globin protein sequences constructed by our method without considering the point distribution information.

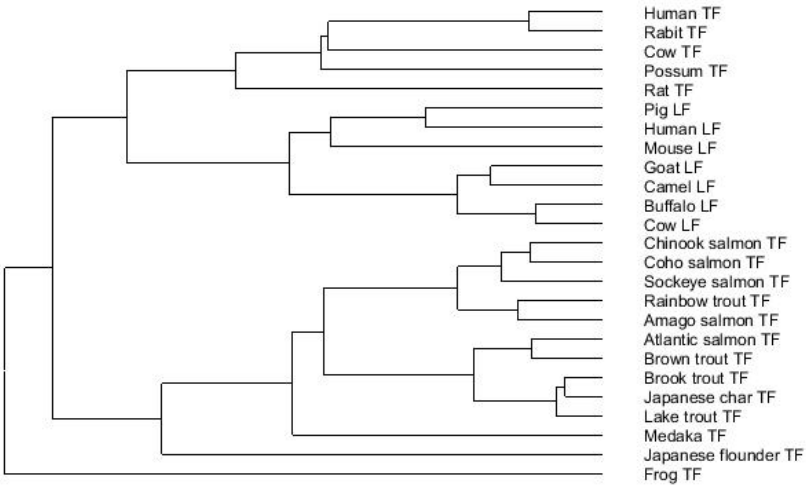

Figure S24. Phylogenetic tree of the 25 TFs constructed by our method without considering the point distribution information.
